# Longitudinal associations among mindfulness, cognitive reappraisal, expressive suppression, non-suicidal self injury, and suicidal ideation among Chinese adolescents

**DOI:** 10.3389/fpsyt.2025.1632526

**Published:** 2025-09-08

**Authors:** Guangzhe Frank Yuan, Shuang Zhong, Caimeng Liu, Zhaoxin Guo

**Affiliations:** ^1^ Sichuan Rural Education Development Research Center, School of Education Science, Leshan Normal University, Leshan, China; ^2^ Mental Health Guidance Center, Ningbo University, Ningbo, China

**Keywords:** mindfulness, cognitive reappraisal, expressive suppression, NSSI, suicidal ideation

## Abstract

**Introduction:**

Prior research has shown that mindfulness may be as a protective factor against self-injurious behaviors and suicidal ideation. Yet, data on the associations between mindfulness, cognitive reappraisal, expressive suppression, non-suicidal self injury (NSSI), and suicidal ideation are limited.

**Method:**

This study attempted to examine the potential mediational relationship between these variables using a two-wave follow-up data (T1 and T2; 6-month intervals) from a sample of 548 Chinese adolescents (*M*_age_=16.10, SD=1.61, age range: 12–19 years; 51.1% boys).

**Results:**

Results indicated that mindfulness at T1 was negatively associated with NSSI and suicidal ideation at T2. Cognitive reappraisal served as a mediator in the mindfulness-suicidal ideation relationship, while both cognitive reappraisal and expressive suppression functioned as parallel mediators in the mindfulness-NSSI relationship. Additionally, cognitive reappraisal and NSSI sequentially mediated the relationship between mindfulness and suicidal ideation.

**Conclusions:**

These findings highlight the importance of fostering mindfulness and adaptive emotion regulation strategies in NSSI and suicide prevention programs for adolescents. Interventions that enhance mindfulness and promote cognitive reappraisal, while reducing reliance on expressive suppression, may effectively diminish self-injurious behaviors and suicidal thoughts among adolescents.

## Introduction

Adolescence represents a critical developmental period, during which adolescents undergo significant physical, emotional, and social changes. During this period, many adolescents exhibit heightened emotional reactivity and vulnerability to mental health issues, including non-suicidal self-injury (NSSI) and suicidal ideation (1, 2). NSSI, defined as the deliberate, self-inflicted destruction of body tissue without suicidal intent (3), affects approximately 16% of adolescents globally (4). Additionally, suicidal ideation, characterized by thoughts about ending one’s life, is also prevalent among adolescents, with estimates ranging from 11.6% to 28.4% (5).

The relationship between NSSI and suicidal ideation has been well-documented in the existing literature. Individuals who engage in NSSI are at increased risk for suicidal thoughts and behaviors (6, 7). Although NSSI and suicidal ideation are distinct phenomena, they frequently co-occur and share common risk factors (8). Given the significant personal and societal costs associated with these behaviors, it is imperative to identify protective factors and elucidate the mechanisms through which they operate in order to develop effective prevention and intervention strategies.

One such protective factor that has attracted increasing attention in recent years is mindfulness. Mindfulness, defined as the awareness that emerges through paying attention on purpose, in the present moment, and without judgement to the unfolding of experience moment by moment (9), has been associated with various positive mental health outcomes. The extant literature indicates that mindfulness-based interventions can mitigate psychological distress in adolescents (10, 11). Furthermore, the emerging evidence suggests that mindfulness may function as a protective factor against self-injurious behaviors and suicidal ideation (12, 13).

The focus on emotion regulation strategies as potential mechanisms linking mindfulness to NSSI and suicidal ideation is grounded in substantial theoretical and empirical evidence. First, adolescence is characterized by heightened emotional intensity and limited emotion regulation capacity (14), making emotion regulation a particularly critical factor during this developmental period. Second, deficits in emotion regulation have been consistently identified as key risk factors for both NSSI and suicidal ideation (15, 16). Third, mindfulness practice has been shown to enhance emotion regulation capacities by increasing emotional awareness, acceptance, and flexibility in responding to emotional experiences (17). Therefore, examining emotion regulation strategies as mediating mechanisms provides a theoretically coherent pathway for understanding how mindfulness may protect against self-injurious behaviors.

Two primary emotion regulation strategies that have been extensively studied in relation to both mindfulness and self-injurious behaviors are cognitive reappraisal and expressive suppression (18, 19). Cognitive reappraisal involves a change in the way one thinks about a situation, which serves to alter its emotional impact. In contrast, expressive suppression involves the inhibition of outward emotional expression. Generally, cognitive reappraisal is considered a more adaptive strategy associated with better psychological outcomes, whereas expressive suppression is often viewed as less adaptive and linked to poorer mental health (20). Research has demonstrated that individuals who engage in NSSI often exhibit difficulties with adaptive emotion regulation, relying more heavily on maladaptive strategies such as expressive suppression and less on adaptive strategies like cognitive reappraisal (21). Similarly, studies have found that individuals with suicidal ideation show reduced use of cognitive reappraisal and increased use of expressive suppression (22).

Mindfulness practices have been demonstrated to facilitate cognitive reappraisal abilities (23) and diminish the reliance on expressive suppression (24). By fostering a nonjudgmental awareness of present-moment experiences, mindfulness may facilitate more adaptive appraisals of stressful situations and reduce the tendency to suppress emotions. These changes in emotion regulation strategies may, in turn, lead to a reduction in the engagement in NSSI and lower levels of suicidal ideation.

The theoretical framework of this study is based on the mindfulness-to-meaning theory (25) and the process model of emotion regulation (26). The mindfulness-to-meaning theory (25)proposes a specific mechanism by which mindfulness practice leads to improved well-being: through enhanced positive reappraisal of stressful events, which reduces negative affect and promotes meaning-making. According to this theory, mindfulness facilitates a metacognitive shift that allows individuals to disengage from automatic, habitual responses to stress and instead engage in more adaptive cognitive reappraisal processes. The process model of emotion regulation complements this framework by suggesting that different emotion regulation strategies can be employed at various points in the emotion-generative process, with distinct consequences for emotional experience and expression. Cognitive reappraisal, as an antecedent-focused strategy, occurs early in the emotion-generation process and is generally more effective than response-focused strategies like expressive suppression. Together, these theories suggest that mindfulness may influence NSSI and suicidal ideation by enhancing the use of adaptive emotion regulation strategies (cognitive reappraisal) while reducing reliance on maladaptive strategies (expressive suppression).

Furthermore, the interpersonal theory of suicide (27) suggests that engagement in NSSI may increase an individual’s capability for suicide by habituating them to self-inflicted pain and fear of death. Existing empirical studies have demonstrated this relationship. For example, using a sample of 565 Chinese adolescents, Giletta et al. (28) found that individuals who exhibited a chronically high level of NSSI were at the highest risk of exhibiting a chronically high level of suicidal ideation. Similar results were found in a study by Liu et al. (29) in a sample of 2,716 Chinese adolescents. They found that NSSI positively predicted suicidal ideation. Thus, we propose that NSSI may mediate the relationship between emotion regulation strategies (i.e., cognitive reappraisal and expressive suppression) and suicidal ideation.

The present study addresses several important research gaps and innovations. First, while previous research has examined mindfulness and emotion regulation strategies separately in relation to NSSI and suicidal ideation, few studies have integrated these constructs within a comprehensive longitudinal mediation model. Second, most existing research has been cross-sectional, limiting our understanding of the temporal relationships among these variables. Third, there is limited research examining these relationships in Chinese adolescent populations, despite the significant prevalence of NSSI and suicidal ideation in this cultural context. Fourth, this study is among the first to examine the sequential mediating roles of emotion regulation strategies and NSSI in the relationship between mindfulness and suicidal ideation.

**Figure 1:**
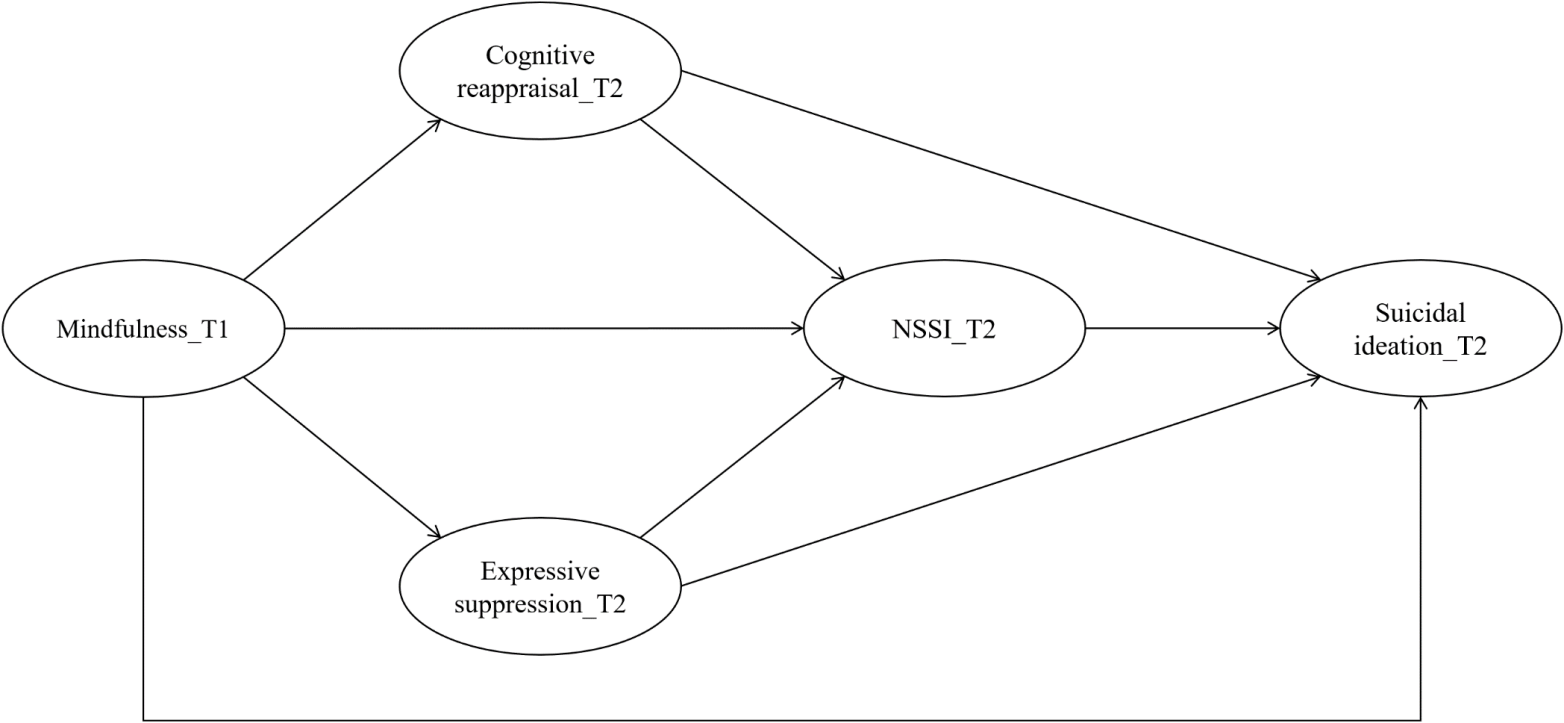
Hypothesized mediation model. T1= wave 1, September 2023; T2= wave 2, March 2024; NSSI, nonsuicidal self-injury.

Drawing on the aforementioned theoretical frameworks and empirical studies, our study aims to investigate the prospective relationship between mindfulness and NSSI and suicidal ideation among adolescents. Furthermore, we seek to examine whether this relationship is indirectly affected by two hypothesized variables, namely cognitive reappraisal and expressive suppression. Specifically, we hypothesize that: (1) mindfulness at baseline would be negatively associated with NSSI and suicidal ideation over time; (2) the relationship between mindfulness and NSSI would be mediated by cognitive reappraisal and expressive suppression; and (3) the relationship between mindfulness and suicidal ideation would be sequentially mediated by cognitive reappraisal, expressive suppression, and NSSI, as illustrated in **Figure 1**.

## Material and methods

### Participants and procedure

This study employed data from an ongoing longitudinal investigation examining the prevalence and underlying mechanisms of NSSI and suicide-related behaviors among Chinese adolescents. A convenience sampling method was employed for the recruitment of participants from two middle schools in southwest China. The study commenced with an initial approach to 701 students across 20 classes (10 from each school) for the purpose of soliciting their participation. The data were collected in two waves, with a six-month interval between them: Time 1 (T1) and Time 2 (T2). T1 assessment was conducted during the fall semester (September 25, 2023 – October 13, 2023), valid responses: N=698 (49.8% female; Mage=15.12, SD=1.90), response rate: 99.6%; T2 assessment was conducted during the spring semester (March 9, 2024 – March 22, 2024), valid responses: N=548 (49.1% female; Mage=16.10, SD=1.61), retention rate: 78.5%. The final analytical sample consisted of 548 participants who completed the assessments at both time points. The age range of participants was 12–19 years. The results of the attrition analysis indicated that there were no statistically significant differences between the participants who completed both waves and those who was lost to follow-up after T1 with respect to key demographic variables or study measures (all *p*s >.05).

The choice of a 6-month interval was informed by previous longitudinal research on adolescent NSSI and suicidal ideation, which suggests that meaningful changes in these behaviors can occur within relatively short timeframes during adolescence (7, 28). Additionally, this interval aligns with the academic calendar structure, allowing for assessments during stable periods of the school year while avoiding major academic transitions that could confound the results.

The Research Ethics Committee of the School of Education Science, first author’s university granted approval for this study. Prior to providing informed consent, participants and their parents were provided with comprehensive information about the research objectives, methodology, and data protection protocols. Participants were informed that they could withdraw from the study at any time without consequence. At each wave, participants completed a 40-minute paper-and-pencil self-report questionnaire. As compensation for their involvement, participants received a gift valued at approximately 10 CNY (US$2) per wave.

### Measures

#### Suicidal ideation

Five items extracted from the 21-item Beck Scale for Suicide Ideation (30) were used to assess suicidal ideation at both waves, as suggested by previous studies (29, 31). These items were rated on a 3-point scale from 0 (no) to 2 (moderate to strong). Sample items include, “I wish to die” and “I desire to make a passive suicide attempt”. A higher total score represents a higher level of suicidal ideation. In this study, the scale demonstrated great internal consistency (Cronbach’s α at T1 and T2 were.83 and.80, respectively).

#### NSSI

The Chinese version of Deliberate Self-harm Inventory (DSI; 32) was used to assess NSSI at both waves. Based on Gong et al. (33)’s recommendation, seven NSSI behaviors were included in the measurements, namely self-cutting, burning, scratching skin, inserting objects to the nail or skin, biting, punching, and banging the head or other parts of the body against the wall. These items were rated on a 4-point scale from 1 (never) to 4 (six times or more). Higher total score of the scale represents more frequent NSSI behaviors. In this study, the questionnaire showed great internal consistency (Cronbach’s α at T1 and T2 were.82 and.84, respectively).

#### Mindfulness

The Chinese version of Child and Adolescent Mindfulness Measure (CAMM; 34) was used to assess mindfulness at both waves. It consists of ten items, each rated on a 5-point scale from 0 (never true) to 4 (always true). Example of items include, “I get upset with myself for having certain thoughts” and “I push away thoughts that I don’t like.”. All items were reverse scored. Higher total score of the scale represents higher level of mindfulness. In this study, this scale demonstrated great internal consistency (Cronbach’s α at T1 and T2 were.87 and.89, respectively).

#### Cognitive reappraisal and expressive suppression

The Chinese version of the Emotion Regulation Questionnaire-Short Form (ERQ-S; 35) was used to assess cognitive reappraisal and expressive suppression. It consists of six items that divided into two subscales: cognitive reappraisal (item 1, 3, 5) and expressive suppression (item 2, 4, 6). All items were rated on a 7-point scale from 1 (strongly disagree) to 7 (strongly agree). Sample items include, “I control my emotions by changing the way I think about the situation I’m in” and “I keep my emotions to myself”. Higher total score of the scale represents higher level of cognitive reappraisal and expressive suppression. In this study, both subscales demonstrated great internal consistency (Cronbach’s α of cognitive reappraisal at T1 and T2 were.83 and.83, respectively; Cronbach’s α of expressive suppression at T1 and T2 were.84 and.83, respectively).

### Data analysis

SPSS 24.0 and Amos 21.0 were used to perform the preliminary analyses (e.g., descriptive statistics and correlation analyses) and mediation model analysis. The final sample (*n*=548) exhibited no missing data for the primary study variables (i.e., mindfulness, cognitive reappraisal, expressive suppression, NSSI, and suicidal ideation). *T*-test analyses were conducted to evaluate the existence of significant differences between participants who completed both waves of the study (*n*=548) and those who were lost to follow-up (*n*=150) with respect to all study variables. The results demonstrated that there were no statistically significant differences between the two groups on the main study variables (*p*s >.05).

A structural equation modeling (SEM) approach was utilized to examine the longitudinal mediation relationships among the study variables. A latent variable was constructed for each construct. To enhance the accuracy, validity, and interpretability of the model, cognitive reappraisal and expressive suppression at T1, NSSI at T1, suicidal ideation at T1, age, and gender were included as covariates. Age and gender were controlled as they are well-established demographic factors associated with both emotion regulation strategies and self-injurious behaviors in adolescent populations (36, 37). Controlling for T1 levels of the outcome variables allows for examination of changes over time while accounting for baseline individual differences. Model fit was improved by examining modification indices and incorporating additional paths as necessary. The statistical significance of hypothesized mediation effects was evaluated using 5000 bootstrapped samples in Amos. Parameters were estimated using maximum likelihood estimation. Model fit was assessed using the following indices: Chi-squared (*χ* ^2^), comparative fit index (CFI), Tucker-Lewis index (TLI), root mean square error of approximation (RMSEA), and standardized root mean square residual (SRMR). Acceptable model fit was determined by the following criteria (38): *χ* ^2^ to degree of freedom ratio less than 5.0, CFI and TLI values greater than 0.90, and RMSEA and SRMR values less than 0.08.

## Results

### Descriptive findings

**Table 1** displays the descriptive statistics and intercorrelations among the study variables. Significant correlations were observed between mindfulness at T1, cognitive reappraisal at T1 and T2, expressive suppression at T1 and T2, NSSI at T1 and T2, and suicidal ideation at T1 and T2. The majority of scale scores demonstrated moderate intercorrelations (*p*s <.01), with some exceptions. Expressive suppression at T1 exhibited a small but significant correlation with NSSI at T2 (*r*=.11, *p* <.05) after controlling for age and gender. Expressive suppression at T1 did not significantly correlate with cognitive reappraisal at either T1 or T2 (*p*s >.05). Expressive suppression at T2 was not significantly related to cognitive reappraisal at T1 (*p* >.05).

**Table 1:** Means, standard deviations, and correlations among study variables (*n*=548).

	M±SD	1	2	3	4	5	6	7	8	9
1. Mindfulness_T1	28.08±7.88	–	.17^***^	-.20^***^	.12^**^	-.17^***^	-.37^***^	-.24^***^	-.43^***^	-.28^***^
2. Cognitive reappraisal_T1	14.68±3.77	.16^***^	–	-.01	.30^***^	-.05	-.25^***^	-.15^**^	-.33^***^	-.19^***^
3. Expressive suppression _T1	12.84±4.43	-.21^***^	.00	–	-.00	.39^***^	.16^***^	.11^*^	.26^***^	.19^***^
4. Cognitive reappraisal _T2	14.10±3.83	.11^*^	.31^***^	.01	–	.23^***^	-.13^**^	-.24^***^	-.18^***^	-.22^***^
5. Expressive suppression_T2	13.19±4.28	-.18^***^	-.04	.39^***^	.24^***^	–	.12^**^	.14^**^	.22^***^	.26^***^
6. NSSI_T1	8.45±2.78	-.38^***^	-.23^***^	.17^***^	-.11^*^	.13^**^	–	.47^***^	.61^***^	.37^***^
7. NSSI_T2	8.52±2.86	-.25^***^	-.13^**^	.13^**^	-.22^***^	.15^***^	.48^***^	–	.38^***^	.63^***^
8. Suicidal ideation_T1	1.53±2.01	-.44^***^	-.32^***^	.27^***^	-.17^***^	.23^***^	.60^***^	.39^***^	–	.53^***^
9. Suicidal ideation_T2	1.49±1.90	-.29^***^	-.18^***^	.20^***^	-.20^***^	.27^***^	.37^***^	.63^***^	.54^***^	–

Left/bottom triangle is the Pearson’s correlations of main study variables, right/top triangle is the partial correlations of main study variables adjusted by sex and age. T1= wave 1, September 2023; T2= wave 2, March 2024; NSSI, nonsuicidal self-injury; ^***^ *p* <.001, ^**^ *p* <.01, ^*^ *p* <.05.

### Mediation results

**Figure 2:**
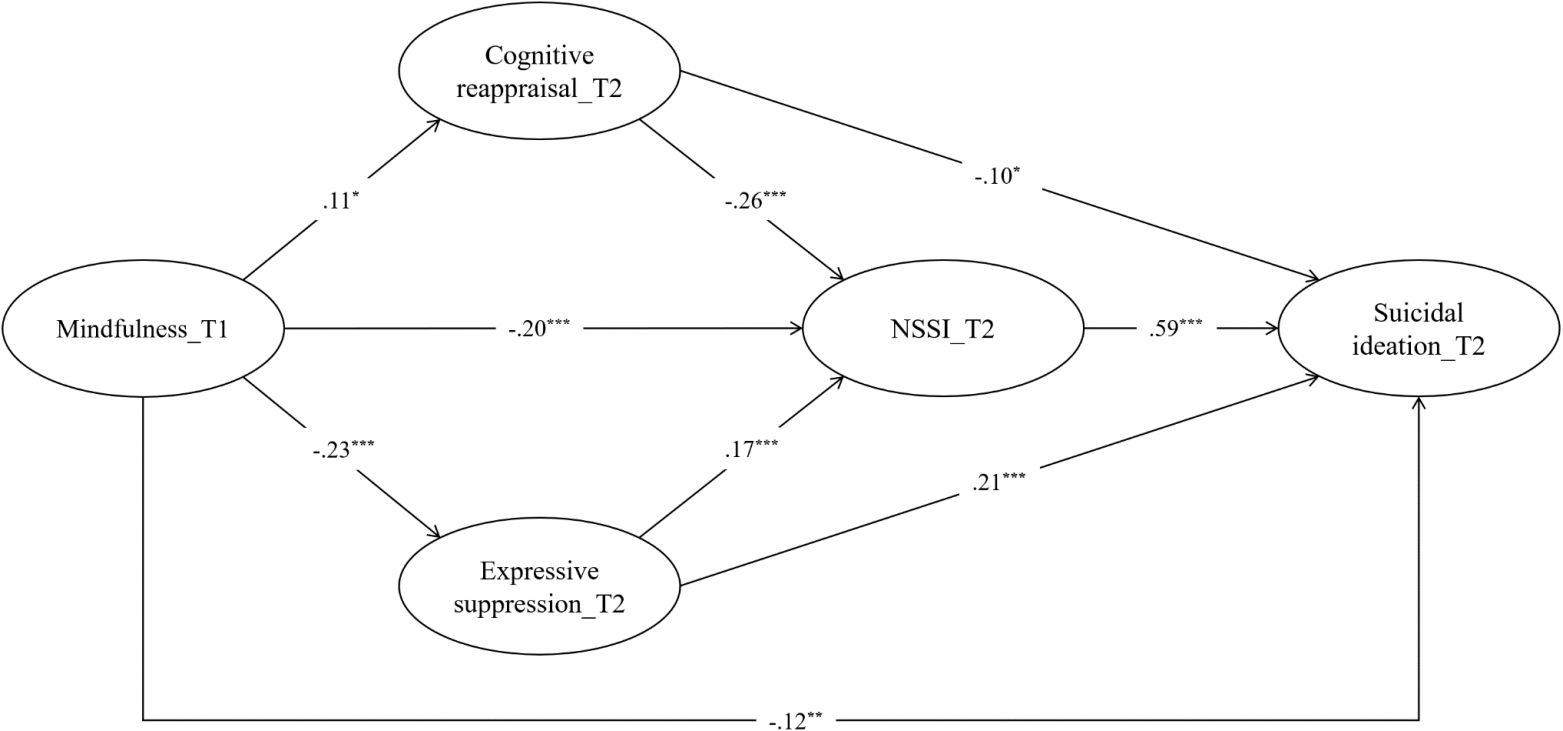
Examined mediation model with standardized coefficients. For simplicity, covariates (i.e., cognitive reappraisal, expressive suppression, NSSI, and suicidal ideation at T1, age, and gender) and factor loadings are not displayed. T1= wave 1, September 2023; T2= wave 2, March 2024; NSSI, nonsuicidal self-injury; ^***^ *p* <.001, ^**^ *p* <.01, ^*^ *p* <.05.

**Figure 2** illustrates the path coefficients of the mediation model. The overall model fit of the hypothesized model was acceptable, *χ* ^2^/*df*=2.73, CFI=0.91, TLI=0.90, SRMR=0.06, RMSEA (90% CI)=0.06 (0.05 – 0.06). Mindfulness was positively related to cognitive reappraisal (*β*=.11, *p* <.05) and negatively related to expressive suppression (*β*=-.23, *p* <.001), NSSI (*β*=-.20, *p* <.001), and suicidal ideation (*β*=-.12, *p* <.01). Cognitive reappraisal was negatively related to NSSI (*β*=-.26, *p* <.001) and suicidal ideation (*β*=-.10, *p* <.05). Expressive suppression was positively related to NSSI (*β*=.17, *p* <.001) and suicidal ideation (*β*=.21, *p* <.001). NSSI was positively associated with suicidal ideation (*β*=.59, *p* <.001).

Furthermore, to further elucidate the indirect effects of cognitive reappraisal and expressive suppression, we employed the bias corrected bootstrap test. The significance of the indirect path coefficient was determined by examining its 95% confidence interval (CI); if this interval did not include 0, the indirect path coefficient was considered significant. As shown in **Table 2**, most indirect paths were significant except for one path - mindfulness->expressive suppression->NSSI->suicidal ideation (*β*=-.01, 95% CI [-.02,.00]), where the confidence interval included zero.

**Table 2:** Bias-corrected bootstrap test on standardized indirect effects.

Paths	*β*	Standardized 95% CI	
		Low	High
MIND_T1 – CR_T2 – SI_T2	-.01^**^	-.03	-.00
MIND_T1 – ES_T2 – SI_T2	-.02^**^	-.04	-.01
MIND_T1 – NSSI_T2 – SI_T2	-.12^***^	-.17	-.07
MIND_T1 – CR_T2 – NSSI_T2 – SI_T2	-.01^*^	-.02	-.00
MIND_T1 – ES_T2 – NSSI_T2 – SI_T2	-.01	-.02	.00

These results were computed after controlling for the covariates (age, gender, and T1 levels of cognitive reappraisal, expressive suppression, NSSI, and suicidal ideation). T1= wave 1, September 2023; T2= wave 2, March 2024; MIND, mindfulness; CR, cognitive reappraisal; ES, expressive suppression; NSSI, nonsuicidal self-injury; SI, suicidal ideation; ^***^ *p* <.001, ^*^ *p* <.05, **p <.01.

## Discussion

To our knowledge, this is the first longitudinal study to explore the relationship between mindfulness, cognitive reappraisal, expressive suppression, NSSI, and suicidal ideation among adolescents. The findings of this study support our hypotheses regarding the protective role of mindfulness against NSSI and suicidal ideation over time. Specifically, our results revealed several significant mediation pathways: (1) cognitive reappraisal mediated the relationship between mindfulness and suicidal ideation, (2) both cognitive reappraisal and expressive suppression served as parallel mediators in the mindfulness-NSSI relationship, and (3) cognitive reappraisal and NSSI sequentially mediated the relationship between mindfulness and suicidal ideation. Notably, the sequential mediation pathway through expressive suppression and NSSI to suicidal ideation was not statistically significant.

In our examined model, we found a significant direct relationship between mindfulness at baseline and NSSI and suicidal ideation over time. This finding highlights the potential protective role of mindfulness against self-injurious behaviors and suicidal thoughts in adolescents. Our results align with previous research suggesting that mindfulness-based interventions can mitigate self harm behaviors in youth (39). The longitudinal design of our study extends these findings by demonstrating that the benefits of mindfulness persist over time. This protective effect may be attributed to mindfulness’s capacity to enhance emotional awareness and regulation, as proposed by Garland et al. (25) in their mindfulness-to-meaning theory. It is possible that by fostering a non-judgmental awareness of present-moment experiences, mindfulness may help adolescents develop more adaptive responses to emotional distress, thereby reducing the likelihood of engaging in NSSI or experiencing suicidal ideation (40).

Our findings reveal a significant serial mediational path, whereby cognitive reappraisal and NSSI serially affected the association between mindfulness and suicidal ideation over time. The complex pathway posits that mindfulness may exert an influence on suicidal ideation through its effects on cognitive reappraisal and subsequent engagement in NSSI. One potential explanation is that mindfulness enhances an individual’s capacity to reappraise adverse circumstances (41, 42), which may result in reduced emotional distress and a diminished propensity to engage in NSSI as a coping strategy. This, in turn, may serve to reduce the risk of suicidal ideation. This interpretation is consistent with the mindfulness-to-meaning theory (25), which postulates that mindfulness enables a constructive reappraisal of challenging circumstances. Moreover, our findings lend support to the notion that NSSI may act as a conduit to suicidal ideation, as postulated by the interpersonal theory of suicide (27). It can be inferred that the reduction in NSSI through improved cognitive reappraisal may thus indirectly result in a decrease in suicidal ideation. These findings extend those of Chesin et al. (43), who suggested that mindfulness-based interventions may reduce both NSSI and suicidal ideation. This study elucidates a potential mechanism through which this effect occurs.

Additionally, although the mediation path where expressive suppression and NSSI indirectly affected the relation of mindfulness and suicidal ideation was not statistically significant, the mediation pathway by which expressive suppression mediated the relation of mindfulness and suicidal ideation was significant. It seems reasonable to posit that individuals who are more mindful may be more aware of their emotional experiences and less likely to engage in expressive suppression (24), which in turn reduces the risk of suicidal ideation (44). Mindfulness enables individuals to observe and accept their emotions without judgment, thereby facilitating the development of healthier emotion regulation strategies and a reduction in the need for expressive suppression (45). Consequently, a reduction in the use of expressive suppression may result in a decreased risk of suicidal ideation, as individuals are more likely to process and cope with their emotions in an adaptive manner (22). This indirect relationship underscores the necessity of addressing expressive suppression in mindfulness-based interventions for suicide prevention (46). By cultivating mindfulness skills, individuals can develop a more accepting and non-judgmental stance towards their emotions, reducing their reliance on maladaptive emotion regulation strategies like expressive suppression, and ultimately decreasing their risk of suicidal ideation (47).

This study has several important limitations that warrant careful consideration. First, although the study employed a two-wave longitudinal design, the six-month interval between assessments may not be sufficient to capture the long-term effects of mindfulness on NSSI and suicidal ideation, as these behaviors may develop or change over extended periods (48). The relatively short timeframe limits our ability to examine whether the observed protective effects of mindfulness persist over longer periods and whether there may be delayed effects that emerge beyond six months. Future research should employ longer follow-up periods with multiple assessment points to better understand the temporal dynamics of these relationships. Second, the study employed self-report measures, which may be susceptible to response bias or social desirability, potentially impacting the accuracy of the data. Given the sensitive nature of the constructs assessed (particularly NSSI and suicidal ideation), participants may have underreported their experiences. Future studies would benefit from incorporating multiple informants (e.g., parents, teachers) or objective behavioral indicators where possible (49, 50). Third, the data were collected from a sample of Chinese adolescents, which may limit the generalizability of the results to other populations, such as adults or individuals from different cultural backgrounds. Cultural factors may influence both the expression of mindfulness and emotion regulation strategies, as well as attitudes toward self-injury and help-seeking behaviors. Cross-cultural replication studies are needed to establish the generalizability of these findings. Fourth, the current model includes T1 predictors as covariates, which controls for baseline levels but does not allow for examination of dynamic or reciprocal relationships between variables over time (51). Cross-lagged panel modeling (CLPM) could provide insights into bidirectional influences between mindfulness, emotion regulation, and suicidal behaviors, but would require additional waves of data collection. Finally, the study did not consider other potential mediators or moderators, such as family functioning, peer support, or trauma exposure, which may influence the relationships among the variables and provide a more comprehensive understanding of the underlying mechanisms (48, 52).

Despite these limitations, the findings of this study have significant theoretical and practical implications. From a theoretical perspective, this study contributes to our understanding of the mechanisms through which mindfulness may protect against self-injurious behaviors and suicidal ideation among adolescents. The findings support and extend the mindfulness-to-meaning theory by demonstrating that mindfulness’ protective effects operate partially through enhanced cognitive reappraisal and reduced expressive suppression. The study also provides empirical support for integrating different theoretical frameworks (mindfulness-to-meaning theory, process model of emotion regulation, and interpersonal theory of suicide) to understand complex mental health phenomena.

From a practical perspective, the findings have direct implications for clinical practice and the design of interventions aimed at preventing and reducing NSSI and suicidal ideation among adolescents. First, the results support the integration of mindfulness-based techniques into prevention and intervention programs, as mindfulness may function as a protective factor against self-injurious behaviors and suicidal ideation (53). Specifically, interventions should focus on developing adolescents’ capacity for present-moment awareness and non-judgmental acceptance of emotional experiences. Second, the results underscore the necessity of focusing on emotion regulation strategies, particularly cognitive reappraisal and expressive suppression, in therapeutic interventions for adolescents engaging in NSSI or experiencing suicidal ideation (15). Interventions should explicitly teach adaptive emotion regulation skills while helping adolescents reduce their reliance on maladaptive strategies. Third, mental health professionals should consider incorporating comprehensive assessments of mindfulness and emotion regulation skills into their work with adolescents at risk for NSSI and suicide, as these may serve as important targets for intervention and indicators of treatment progress.

Future research should address several key directions to advance our understanding of these relationships. First, longitudinal studies with multiple time points and longer follow-up periods are needed to examine the stability and developmental trajectories of these associations. Second, intervention studies should test whether mindfulness-based programs that specifically target emotion regulation skills are more effective than standard mindfulness interventions in reducing NSSI and suicidal ideation. Third, research should examine potential cultural moderators of these relationships, particularly given the growing recognition of cultural factors in mental health. Fourth, studies should investigate the role of additional mediators and moderators, such as social support, family functioning, and trauma exposure, to develop more comprehensive models of risk and protection. Finally, research employing ecological momentary assessment methods could provide insights into the real-time dynamics of mindfulness, emotion regulation, and self-injurious thoughts and behaviors.

## Conclusions

In conclusion, the findings of this study provide evidence for the protective role of mindfulness against NSSI and suicidal ideation among Chinese adolescents, operating through multiple pathways involving emotion regulation strategies. Specifically, mindfulness appears to reduce the risk of NSSI and suicidal ideation by enhancing cognitive reappraisal capabilities and reducing reliance on expressive suppression. The sequential mediation through cognitive reappraisal and NSSI further suggests that improvements in adaptive emotion regulation may reduce self-injurious behaviors, which in turn decreases suicide risk. These findings highlight the importance of integrating mindfulness-based techniques and adaptive emotion regulation training into prevention and intervention programs for adolescents at risk for NSSI and suicide. The study contributes to both theoretical understanding and practical applications in adolescent mental health, while pointing toward important directions for future research to further elucidate these complex relationships.

## Data Availability

The raw data supporting the conclusions of this article will be made available by the authors, without undue reservation.
